# MYCO-TB: the first IVD kit suitable for the digestion and decontamination of extra-pulmonary specimens to detect Mycobacteria

**DOI:** 10.1038/s41598-021-93182-z

**Published:** 2021-07-01

**Authors:** Francesco Bisognin, Giulia Lombardi, Silvia Felici, Paola Dal Monte

**Affiliations:** 1 Department of Experimental, Diagnostic and Specialty Medicine, Alma Mater Studiorum University of Bologna, Bologna, Italy; 2 Microbiology Unit, IRCCS Azienda Ospedaliero-Universitaria di Bologna, Policlinico di S. Orsola, Via Massarenti 9, 40138 Bologna, Italy

**Keywords:** Bacteriology, Infectious-disease diagnostics

## Abstract

Extra-pulmonary mycobacterial infections are characterized by a paucibacillary nature and extra-pulmonary samples consist of different matrices; the processing of these samples requires a high level of manual skills and non-standardized procedures. The aim of this study was to compare the performance of MYCO-TB with MycoPrep on extra-pulmonary samples in terms of Mycobacteria detection, culture contamination and suitability for molecular assay. This prospective study was conducted on 201 extra-pulmonary samples from suspected cases of mycobacterial infection. Specimens were divided into two equal aliquots; one was decontaminated with MYCO-TB the other with MycoPrep. The contamination rate of liquid cultures was significantly different: 2.5% (5/201) for MYCO-TB and 7.5% (15/201) for MycoPrep (*p* = 0.036). At least 1 *Mycobacterium tuberculosis* complex (MTBc) positive culture was detected in 6 specimens treated with MYCO-TB and 8with MycoPrep, without significant differences in times to positivity (TTP) in liquid culture. No Xpert MTB/RIF Ultra invalid results were obtained with samples decontaminated with MYCO-TB. The MYCO-TB kit had greater activity than MycoPrep in the digestion and decontamination of extra-pulmonary specimens for the detection of Mycobacteria, supporting the use of MYCO-TB in this type of sample. Ready-to use reagents, rapid protocol and single-sample formulation of MYCO-TB reduced the level of manual skills required as well as the risk of sample contamination.

## Introduction

Mycobacterial infections, caused by *Mycobacterium tuberculosis* complex (MTBc) and Non-tuberculous Mycobacteria (NTM), are frequently associated with lung disease and most commercial IVD products intended for the detection of Mycobacteria have been designed for respiratory specimens.

The proportion of mycobacterial infections with extra-pulmonary involvement is 29.4% of total TB cases notified in Italy and 22.9% of total TB cases notified in Europe^1^.

Extra-pulmonary mycobacterial infections are characterized by a paucibacillary nature^2^. The gold standard for diagnosis is culture^3^. During the lengthy incubation required for microbiological diagnosis with both respiratory and extra-pulmonary samples, contaminating organisms may overgrow in cultures and prevent detection. Therefore, culture is preceded by gentle but effective digestion and decontamination using a solution based on sodium hydroxide (NaOH) and *N*-acetyl-L-cysteine (NALC)^4^.

The process of digesting and decontaminating extra-pulmonary samples requires a high level of manual skill to prepare reagents, which are often not available in bulk, as well as to process samples with non-standardized procedures.

MYCO-TB is a single sample digesting and decontaminating kit developed by Copan (Italy), with a shorter sample processing time than other commercial kits.

In addition, ready-to-use reagents make it possible to reduce and optimize the manual skills required and avoid errors during sample processing.

In our routine protocol we currently use MycoPrep (Becton Dickinson, BD, USA). The aim of this study was to compare the performance of MYCO-TB with MycoPrep, both based on NALC and NaOH, in terms of Mycobacteria detection and culture contamination in extra-pulmonary samples.

## Materials and methods

### Samples

This prospective study was conducted at the Microbiology Unit of S. Orsola-Malpighi University Hospital, a referral centre for the diagnosis of mycobacterial infections for the metropolitan area of Bologna (Italy).

The study was approved by the Ethics Committee of Area Vasta Emilia Centro (AVEC), Bologna, Italy (Study protocol n.133/2020/Sper/AOUBo). All the experiments were conducted according to the principles expressed in the Declaration of Helsinki.

Two hundred and one extra-pulmonary samples from suspected cases of mycobacterial infections between September and December 2019 were randomly selected and anonymized (numbered from 1 to 201) to assess the decontamination performance of MYCO-TB compared to that of MycoPrep. The sample matrices were as follows: 21 (20%) cavitary fluids, 50 (25%) swabs, 40 (20%) urine, 20 (10%) biopsies, 20 (10%) purulent exudates, 10 (5%) lymph-nodes and 20 (10%) gastric aspirates.

For this comparative study, a volume of 10 ml was required for cavitary fluids and gastric aspirates, 50 ml for urine (centrifuged, washed and suspended in 10 ml of Phosphate buffered saline (0.067 M, pH 6.8, PBS), while for the remaining biological samples (swab, biopsy, purulent exudate and lymph-node) the volume was adjusted to 10 ml with PBS. Before decontamination, all samples were analysed by acid-fast microscopic examination using Ziehl–Neelsen staining and divided into two 5 ml aliquots, which were decontaminated with MYCO-TB or MycoPrep in parallel, as described below.

### Decontamination with MycoPrep (Becton Dickinson)

Each MycoPrep kit contains NaOH solution and a sealed glass ampule of lyophilized NALC, to preserve its mucolytic activity^4^.

5 ml of NALC-NaOH solution were added to an equivalent volume of sample. After vortexing for 15 s, decontamination continued at room temperature for 23 min.

PBS was added to bring the volume up to 45 ml and mixed by inversion to neutralize the decontamination reaction.

After centrifugation at 3000*g* for 15 min the supernatant was discarded and the bacterial pellet resuspended with 2 ml of PBS.

### Decontamination with MYCO-TB (Copan)

Each MYCO-TB kit consists of 4 tubes with different coloured caps filled with ready-to-use reagents formulated for single sample use:

1. Reaction tube: a sterile graduated empty tube with a capacity of 50 ml suitable for centrifugation of up to 14,000*g*;
2. MYCO-TB solution tube: a tube containing 5 ml of the fluidification and decontamination solution, composed of sodium hydroxide (NaOH) and *N*-acetyl-L-cysteine (NALC);
3. Neutralizing tube: containing 45 ml of neutralizing solution, a buffered saline solution to neutralize the MYCO-TB reagents;
4. Resuspension tube: filled with 2 ml of buffered saline solution for pellet resuspension after centrifugation.

The neutralizing solution and resuspension reagent are both composed of Sodium phosphate, Sodium chloride and Potassium phosphate.

5 ml of specimen was transferred to the reaction tube, and 5 ml of MYCO-TB reagent was added. The tube was vortexed for 30 s and left at room temperature for 5 min.

Neutralizing solution was added to the reaction tube to a volume of 50 ml and mixed by inversion.

The reaction tube was centrifuged at 3300*g* for 5 min and the supernatant discarded. The sediment was resuspended in 2 ml of resuspension solution.

### Xpert MTB/RIF Ultra

Samples strongly suspected of MTBc infection were analysed by Xpert MTB/RIF Ultra (Ultra, Cepheid, USA). For each decontamination system 500 μl of sample were mixed with Sample Reagent (containing NaOH and isopropanol) in a 1:3 ratio for 15 min at room temperature, poured into a single-use disposable cartridge and subsequently loaded onto the GeneXpert module according to the manufacturer’s instructions^5^. The system automatically interpreted all results from fluorescent signals into the following categories: invalid, if PCR inhibitors were detected with amplification failure, negative or positive. Positive results were divided into 5 categories depending on bacterial load: the relationship between the *rpo*B Ct value and input CFU allows samples to be classified as “high,” “medium, “low,” and “very low” and to define strains as susceptible or resistant to Rifampicin depending on the detection of mutations in the *rpo*B gene, while the “trace” category identifies the paucibacillary samples which are IS6110/IS1081 positive but *rpo*B negative, with indeterminate Rifampicin susceptibility.

### Mycobacteria culture and identification

Lowenstein–Jensen (LJ) solid media with and without antibiotic mix (PACT, BD) were inoculated with 250 µl of decontaminated samples; liquid media Mycobacteria Growth Indicator Tube (MGIT, BD) were inoculated with 0.8 ml of supplement (PANTA, BD) and 0.5 ml of decontaminated sample. LJ agar slants were incubated at 37 °C; MGIT tubes were loaded into the BACTEC MGIT 960, an automated mycobacterial detection system.

Solid and liquid cultures were considered negative if no Mycobacteria were isolated after 42 days of incubation. Samples were considered positive if mycobacterial growth was detected in at least one of the three different media (LJ, LJ PACT, MGIT).

Positive cultures were identified as MTBc by Ultra, or as Non-Tuberculous Mycobacteria (NTM) by GenoType CM (Hain Lifescience, Germany).

Mycobacteria time to positivity (TTP) in liquid culture was defined as the number of days from MGIT incubation into BACTEC MGIT 960 to positive culture result, using Epicenter software (BD).

Culture contamination was defined as bacterial or yeast growth preventing Mycobacteria isolation; rates of both solid and liquid culture contamination were calculated.

### Statistical analysis

Student’s t-test was used to compare the TTP in liquid culture and Ultra IS1081/IS6110 probe Ct values of MYCO-TB and MycoPrep-treated MTBc-positive specimens. The rates of culture contamination of samples treated with MYCO-TB and MycoPrep were compared using the chi-square test. Cohen’s κ statistics were calculated to assess agreement between the two decontamination methods.

Statistical analysis was performed using GraphPad Prism version 8.0.1 (San Diego, CA, USA). Statistical significance was set at *p* < 0.05.

## Results

### Culture contamination

The overall proportions of contamination of both liquid and solid media, defined as the total failure to isolate Mycobacteria, were 1.5% for MYCO-TB and 2.5% for MycoPrep.

The contamination rate of liquid cultures was significantly different: 2.5% (5/201) for MYCO-TB and7.5% (15/201) for MycoPrep (*p* = 0.036). Liquid culture of 10 samples (5 urine and 5 purulent samples) were negative with MYCO-TB, whereas treatment with MycoPrep did not prevent contamination; in these samples Gram-negative bacteria were isolated in 7 cases and Gram-positive in 3.

The contamination rate of solid media was similar for both systems: 8.5% (17/201) and 6.0% (12/201) on LJ; 3.5% (7/201) and 4.0% (8/201) on LJ PACT for MYCO-TB and MycoPrep respectively.

Table 1 shows the agreement between MYCO-TB and MycoPrep methods in terms of culture contamination: overall agreement was 97.79% with moderate concordance (k = 0.489).

**Table 1:** Agreement between MYCO-TB and MycoPrep methods for culture contamination.

Decontamination method	Contaminated Culture				Uncontaminated culture	Agreement (%)	k
	MYCO-TB and MycoPrep	MYCO-TB only		MycoPrep only	MYCO-TB and MycoPrep		
MGIT	5		0	10	186	95.02	0.481
LJ	9		8	3	181	94.53	0.592
LJ PACT	5		2	3	191	97.51	0.654
Overall (MGIT + LJ + LJ PACT)	2		1	3	175	97.79	0.489

### Mycobacteria detection

Out of 201 extrapulmonary samples, MTBc growth was detected in6 specimens decontaminated with MYCO-TB and in 8 specimens decontaminated with MycoPrep. Smear, culture and Xpert results for Myco-TB and MycoPrep decontamination systems are reported in Table 2. No significant difference was observed between average TTP of MTBc in liquid culture decontaminated with MYCO-TB (16.1 ± 8.0 days) and MycoPrep (19.3 ± 8.2 days) (*p* = 0.56).

**Table 2:** Microbiological characteristic of MTBc-positive samples obtained after decontamination with (a) Myco-TB and (b) MycoPrep.

n	Sample type	Smear	MGIT	TTP*	LJ^°^	LJ + PACT^°^	Xpert load	Ct^#^
**(a) Myco-TB**								
91	Cavitary fluid	Neg	Pos	28.75	Neg	Neg	Low	19.8
120	Biopsy	Neg	Pos	17.29	Neg	Neg	Low	18.3
144	Cavitary fluid	Neg	Pos	16.04	1	Neg	Neg	
145	Gastric aspirate	Neg	Pos	11.54	20	18	Low	22.2
155	Cavitary fluid	Neg	Neg		Neg	Neg	Neg	
163	Gastric aspirate	Neg	Neg		Neg	Neg	Neg	
185	Gastric aspirate	Neg	Pos	15.71	1	3	Very low	20.2
196	Purulent exudate	Neg	Pos	7.42	> 50	> 50	Low	17.1
**(b) MycoPrep**								
91	Cavitary fluid	Neg	Pos	27.02	Neg	Neg	Low	17.5
120	Biopsy	Neg	Pos	17.09	Neg	Neg	Low	18.3
144	Cavitary fluid	Neg	Neg		1	Neg	Neg	
145	Gastric aspirate	Neg	Pos	14.46	8	3	Low	22.5
155	Cavitary fluid	Neg	Pos	23.04	Neg	Neg	Trace	23.3
163	Gastric aspirate	Neg	Pos	36.58	Neg	Neg	Neg	
185	Gastric aspirate	Neg	Pos	28.13	Neg	Neg	Trace	23
196	Purulent exudate	Neg	Pos	9.21	> 50	> 50	Low	17.4

*TTP = Time to positivity.

^°^number of Mycobacteria colonies. Neg = negative. Pos = positive.

^#^Ct: Cycle threshold values referred to IS6110/IS1081 probes.

No NTM growth was detected in these samples.

### Xpert MTB/RIF Ultra results

Ultra was performed on 49 samples with strongly suspected MTB infection and was valid for all samples tested, decontaminated by both methods.

Myco-TB-treated samples: Ultra was negative in all MTBc-negative culture samples (n = 41) and positive in 5 out of 6 MTBc-positive culture samples.

MycoPrep-treated samples: Ultra was negative in all MTBc-negative culture samples (n = 41) and positive in 6 out of 8 MTBc-positive culture samples.

Ultra IS1081/IS6110 probe mean Ct values were similar with both decontaminating systems: 19.5 for MYCO-TB and 19.7 for MycoPrep.

## Discussion

Culture is considered the gold standard for microbiological diagnosis of mycobacterial infections, but contaminating organisms can limit diagnostic performance, therefore the type of decontamination system could affect Mycobacteria detection^6^.

This is the first study evaluating the performance of MYCO-TB kit, developed by Copan, for sample digestion and decontamination to detect Mycobacteria in extra-pulmonary samples.

Only two studies have been performed on MYCO-TB, both limited to respiratory samples. De Geyter and colleagues, from University Hospital of Brussels, evaluated the performance of MYCO-TB in comparison to Zephiran method on 387 pulmonary specimens^7^. The contamination rates on solid media were 22% with both the Zephiran and MYCO-TB method while in liquid media it was calculated only for the MYCO-TB Kit (4%) as Zephiran method is incompatible with the MGIT system.

Our group compared MYCO-TB to MycoPrep on 162 respiratory samples^8^ reporting the same overall contamination rate in liquid and solid media (1.8%). Furthermore, we showed that extended time of decontamination (5 and 10 min) with MYCO-TB did not affect MTBc detection in terms of TTP in liquid culture and Ultra performance.

In this study we have shown that the MYCO-TB kit has greater activity than MycoPrep digesting and decontaminating extra-pulmonary specimens with an overall contamination rate of 1.5% and 2.5%, respectively. In particular, the decontamination activity of MYCO-TB was significantly higher than MycoPrep in liquid culture, where 10 MycoPrep treated samples (5 urine and 5 purulent samples) did not successfully complete the incubation required, due to highly contaminated biological matrices. We exclude that this difference could be caused by the people conducting the work or a difference in working environments as the two dedicated laboratory technicians performed the experiments simultaneously in the dedicated laboratory of biosafety level 3. The stronger decontamination activity of MYCO-TB kit could have potential downstream clinical benefits for patients with TB or NTM abscesses, where contaminating organisms present in highly purulent samples might prevent mycobacterial detection.

On MTBc-positive cultures detected by both decontaminating kits, there was no significant difference in time to positivity (16.1 days for MYCO-TB and 19.3 for MycoPrep) in liquid culture or forULTRA IS1081/IS6110 probe Ct values (19.5 for MYCO-TB and 19.7 for MycoPrep).

However, 2 out of 8 MTBc-positive cultures, a cavitary fluid (sample number 155) and a gastric aspirate (sample number 163), detected after MycoPrep treatment, were not detected after decontamination with MYCO-TB. We can speculate that this could be due to the very low Mycobacteria load in these samples, proven by no growth on solid media, trace and negative Ultra results and a high TTP in MycoPrep-treated samples (23.04 and 36.58 days, respectively). A limit of this prospective study is the small sample size of MTBc-positive samples detected, which prevented us from accurately calculating sensitivity and specificity. However, the low number of MTBc-positive culture detected reflects the epidemiology of extra-pulmonary TB in our setting.

Furthermore, samples decontaminated with MYCO-TB were suitable for molecular assays, i.e., Xpert MTB/RIF Ultra assay.

In conclusion, the ready-to-use reagents, formulation for single-sample and the rapid protocol of MYCO-TB (5 vs. 23 min with MycoPrep) made it possible to optimize manual skills, avoid errors and reduce the risk of contamination during sample processing for Mycobacteria detection.

## Data Availability

The data that support the findings of this study are available from the corresponding author upon reasonable request.
